# A Multicenter, Randomized, Double-Blind, Placebo-Controlled Trial to Investigate the Effects of Kamishoyosan, a Traditional Japanese Medicine, on Menopausal Symptoms: The KOSMOS Study

**DOI:** 10.1155/2021/8856149

**Published:** 2021-02-26

**Authors:** Kiyoshi Takamatsu, Mariko Ogawa, Satoshi Obayashi, Takashi Takeda, Masakazu Terauchi, Tsuyoshi Higuchi, Kiyoko Kato, Toshiro Kubota

**Affiliations:** ^1^Department of Obstetrics and Gynecology, Tokyo Dental College Ichikawa General Hospital, Chiba, Japan; ^2^Department of Obstetrics and Gynecology, Dokkyo Medical University, Tochigi, Japan; ^3^Division of Women's Health, Research Institute of Traditional Asian Medicine, Kindai University, Osaka, Japan; ^4^Department of Women's Health, Tokyo Medical and Dental University, Tokyo, Japan; ^5^Department of Nursing Science, Hirosaki University Graduate School of Health Sciences, Aomori, Japan; ^6^Department of Obstetrics and Gynecology, Graduate School of Medicine, Kyushu University, Fukuoka, Japan; ^7^Department of Gynecology, Tokyo Kyosai Hospital, Tokyo, Japan

## Abstract

**Objective:**

The KOSMOS study, a multicenter, randomized, double-blind, placebo-controlled trial, investigated the effects and safety of kamishoyosan (TJ-24), a traditional Japanese medicine, in the treatment of climacteric disorder.

**Methods:**

Japanese women with climacteric disorder were administered a placebo during a 4-week run-in period, after which they were classified as placebo responders (R group) if their score on the modified Questionnaire for the Assessment of Climacteric Symptoms in Japanese Women (m-QACS) with excitability and irritability as the primary outcome improved by ≥ 3 points and as placebo nonresponders (NR group) otherwise. Members of the NR group were randomly allocated to receive either TJ-24 or placebo. After 12 weeks, their m-QACS scores, anxiety and depression, sleep, and overall quality of life (QOL) were compared.

**Results:**

The TJ-24 and placebo arms in the NR group included 20 patients each. The change in the m-QACS scores of members of the NR group for excitability and irritability at 12 weeks versus baseline was –3.1 ± 1.7 in the TJ-24 arm, a significant decrease, but compared with –2.7 ± 2.2 in the placebo arm, no significant difference was between two arms. However, the proportion of participants whose score improved by ≥3 points was significantly higher in the TJ-24 arm. In the subgroup analysis of premenopausal women, the changes in the score for excitability and irritability were significantly larger in the TJ-24 arm. The incidence of adverse drug reactions or adverse events did not differ between the two arms, and no serious events were reported.

**Conclusion:**

Although no significant difference was identified for the primary outcome, a significantly higher proportion of patients who received TJ-24 displayed improvement. Its high level of safety and effects on excitability and irritability in premenopausal women suggest that TJ-24 may be a useful treatment.

## 1. Introduction

Climacteric disorder is a major factor in diminishing quality of life (QOL) starting in perimenopause, and managing its symptoms is an urgent task. Hormone therapy (HT) to augment the level of estrogen, which decreases because of menopause, is known to be effective, and this treatment is provided also in Japan [1]. However, the combined estrogen-progestogen therapy arm of the 2002 Women's Health Initiative study was stopped mainly because of an increased risk of breast cancer [2], HT has been negatively viewed, and nonhormonal therapies have gained popularity. The treatments used in Western countries include selective serotonin reuptake inhibitors and cognitive behavioral therapy [3]. Measures are also required to help perimenopausal women whose estrogen levels remain close to normal.

In Japan, traditional Japanese medicine (Kampo therapy) has long been practiced [4, 5]. Kampo therapy uses combinations of crude natural drugs, and compliance is high. One such formulation, kamishoyosan (TJ-24), has been reported to improve neuropsychiatric symptoms [6, 7], vasomotor symptoms [6], and sleep disturbance [8] in patients with climacteric disorder, and it is widely used. Unfortunately, previous studies have failed to provide evidence of its efficacy [9].

Therefore, we previously conducted a double-blind, randomized, placebo-controlled trial of the efficacy of TJ-24. In that study, although TJ-24 was associated with significant improvements in the frequency of hot flashes, depression, anxiety, and QOL after the start of treatment, no significant difference was identified between the TJ-24 and placebo arms [10]. We concluded that Kampo drug administration elicits a strong placebo effect on menopausal symptoms and that studies focusing on individual symptoms are required.

From these results, we conducted a new double-blind, randomized, parallel-group comparative study to investigate the efficacy and safety of TJ-24, termed the kamishoyosan's effects on some menopausal symptoms (KOSMOS) study. This study adopted a design that included a run-in period of placebo administration and stratification of participants into placebo responders and nonresponders to minimize the placebo effect. In addition, the symptoms observed in climacteric disorder were also subdivided to investigate the effect of TJ-24 in detail; particularly, we focused on excitability and irritability. The results of KOSMOS study are reported.

## 2. Subjects and Methods

### 2.1. Participants

Japanese women aged 40–60 years who complained of climacteric disorder were recruited for the study from the authors' institutions between April 2016 and March 2018. Climacteric disorder was assessed by converting severity scores on the modified Questionnaire for the Assessment of Climacteric Symptoms in Japanese Women (m-QACS) [11] to a 0–10 numerical rating scale, and participants were selected from women with an excitability and irritability score of ≥5 points plus a score of ≥5 points for either sleep disturbance or vasomotor symptoms (i.e., any one of hot flashes, sweating, difficulty in sleeping, or waking up at night).

The exclusion criteria were severe intestinal fragility, poor appetite, nausea or vomiting, serious psychiatric or neurological disorder, existing allergy to Kampo medication, serious comorbidity such as liver disease, heart disease, hematological disease, or malignancy, or any other reason that disqualified the individual concerned in the judgment of the investigator. The concomitant use of female hormones, autonomic neuromodulators, anxiolytics, psychotropics, hypnotics, antidepressants, kallidinogenase formulations, or other Kampo medications was prohibited. Individuals who had used any of these drugs within the previous 4 weeks before enrollment were also excluded. The concomitant use of over-the-counter supplements was permitted, but participants were asked not to start taking a new supplement or to change the dose or administration method during the study period.

### 2.2. Study Protocol

**Figure 1:**
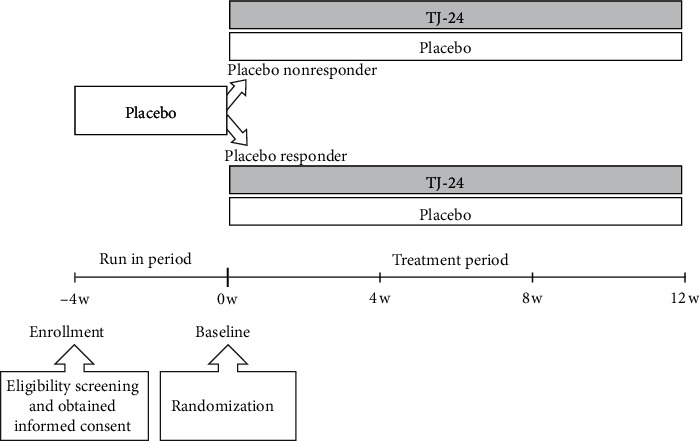
Study design.

As presented in Figure 1, after informed consent was obtained, participants were administered a placebo for a 4-week run-in period. At the end of these 4 weeks, they were classified as placebo responders (R group) if their excitability and irritability score improved by ≥ 3 points and as placebo nonresponders (NR group) otherwise. Blinding and randomization were performed by the investigator responsible for test drug allocation, who used the permuted block method to allocate subjects into the R and NR groups to receive either TJ-24 or an externally indistinguishable placebo. Allocation was performed in the order in which participants were enrolled in each institution.

This study was performed under the Declaration of Helsinki, and the study protocol was approved by the Institutional Review Board of each institution (IRB project No. at Tokyo Dental College, Kindai Univ., Tokyo Medical and Dental Univ., and Hirosaki Univ. were TJ-24-4-1, IV-3-72, 2016–0001, and Rin-1, respectively). This study is registered in JAPIC Clinical Trials Information in Japan (JapicCTI-No.163215), and a summary of this study is available on the JAPIC Clinical Trials Information website (https://www.clinicaltrials.jp/cti-user/trial/Show.jsp).

### 2.3. Sample Size

The target enrollment was 40 participants in the NR group and 20 in the R group, and enrollment was concluded when 40 participants had been enrolled in the NR group. The rationale for this sample size was based on the assumption that a difference in the changing of the m-QACS score between the TJ-24 and placebo arms in the NR group would be ≥4. Therefore, if the standard deviation was 4 and the participants were allocated to the TJ-24 and placebo arms in a 1 : 1 ratio, the sample size required to achieve a detection rate of 80% in a two-tailed *t*-test with *p*=0.05 would be 17 participants in each arm. To account for dropouts, the target enrollment for the NR group was set at 40, with 20 participants each receiving TJ-24 and the placebo. In a study conducted by Plotnikoff et al., the hot flash score improved by <30% after 12 weeks of placebo treatment in 19 of 29 patients, who were thus deemed nonresponders [9], and it was considered that the ratio between the NR and R groups should be 2 : 1. Accordingly, the target enrollment was set at 40 participants in the NR group and 20 in the R group.

### 2.4. Drugs Used

Subjects were administered either kamishoyosan (Tsumura-Kampo Kamishoyosan Extract Granules, Tsumura and Co., Tokyo, Japan) or a placebo having a similar appearance to kamishoyosan, supplied by Tsumura and Co., at a dose of 7.5 g/day divided into three portions and taken before or after each meal. Kamishoyosan was used as a powdered extract obtained by spray-drying a hot extract mixture of the following 10 crude herbs: 13.3% Bupleuri Radix (*Bupleurum falcatum*), 13.3% Paeoniae Radix (*Paeonia lactiflora*), 13.3% Atractylodis Rhizoma (*Atractylodes ovate*), 13.3% Angelicae Radix (*Angelica acutiloba*), 13.3% Hoelen (*Poria cocos*), 8.9% Gardeniae Fructus (*Gardenia jasminoides*), 8.9% Moutan Cortex (*Paeonia suffruticosa*), 6.7% Glycyrrhizae Radix (*Glycyrrhiza uralensis*), 4.4% Zingiberis Rhizoma (*Zingiber officinale*), and 4.4% Menthae Herba (*Mentha arvensis*). The placebo formulation included lactose hydrate, magnesium stearate, cornstarch, dextrin, iron sesquioxide, yellow no. 4 aluminum lake, blue no. 1 aluminum lake, and caramel.

### 2.5. Measured Values

Climacteric disorder symptoms were evaluated using a numerical rating scale of the m-QACS [11]. This self-administered questionnaire assesses the severity of 21 symptoms on a scale of 0 to 10, with 0 being least severe, instead of 0 to 3 in the original version. The scores were checked at each visit, namely, weeks –4, 0 (baseline), 4, 8, and 12. In addition, psychiatric symptoms, namely, anxiety and depression, were assessed using the Hospital Anxiety and Depression Scale (HADS) [12], and sleep disturbance was assessed using the Pittsburgh Sleep Quality Index (PSQI) [13]. Overall QOL was assessed using the SF-8 [14]. The scores of these questionnaires were checked at Weeks 0 (baseline), 4, 8, and 12. Variations of ±1 week were permitted for the investigations at each time point.

To assess safety, the participants underwent two blood tests during the trial, first during the run-in period and then after the 12 weeks of treatment. The major adverse drug reactions to TJ-24 listed in its package insert on the basis of current knowledge are pseudoaldosteronism, myopathy, hepatic impairment, and jaundice, and these were investigated by performing a complete blood count and testing for liver function, kidney function, and electrolytes levels in blood. A physical examination was performed at each hospital visit, during which blood pressure, weight, and edema were checked, and adverse events were reported at the time of occurrence.

### 2.6. Primary and Secondary Outcomes

The primary outcome was the change of the m-QASC score for excitability and irritability in the NR group between the start of the treatment period (week 0) and week 12. Secondary outcomes were the changes in scores for all 21 items in m-QACS, HADS, PSQI, and SF-8 between week 0 and weeks 4, 8, and 12, and subgroup analyses were also conducted with participants stratified according to the baseline menopausal status (≧12 months amenorrhea was defined as menopause, and others were defined as premenopause), body mass index (BMI), the duration of climacteric disorder, and prior hysterectomy/oophorectomy.

### 2.7. Adverse events and Safety

Safety and tolerability were assessed by recording all adverse events and abnormal clinical laboratory test results. Adverse events were defined as “adverse drug reactions” if a causal relationship with the study drug could not be eliminated.

### 2.8. Statistical Analysis

Baseline characteristics were investigated using Student's *t*-test, Fisher's exact test, or Wilcoxon's rank-sum test, depending on the nature of the data. Data obtained from the changes of the m-QACS score, HADS score, PSQI score, and SF-8 score were used to calculate summary statistics for scores at each time point and changes versus baseline. Wilcoxon's signed-rank test was used to analyze the changes in scores from baseline at each time point in each treatment arm, and differences between the arms at each time point and changes from baseline were tested using Wilcoxon's rank-sum test. Comparisons between the arms concerning the distribution of changes in the excitability and irritability score were performed using Fisher's exact test.

The full analysis set (FAS) for the efficacy assessment included participants enrolled in this study, excluding those missing data required for the efficacy analysis and those who clearly failed to take their allocated test drug.

The safety analysis set included all patients who completed the study without restriction.

Adverse events were listed as the lowest-level terms in the ICH Medical Dictionary for Regulatory Activities/Japanese (MedDRA/J) version 21.0. They were tabulated by preferred term and classified by system organ class. The rates of adverse events, adverse drug reactions, serious adverse events, and serious adverse drug reactions in the safety analysis set were tabulated by stratum and arm, and the 95% confidence intervals for their rates were calculated, with Fisher's exact test used for comparisons between arms. Analyses were conducted using SAS 9.4 software (SAS Institute Inc., Cary, NC, USA), with *p* < 0.05 regarded as significant.

## 3. Results

### 3.1. Status of Placebo Response and Baseline Characteristics in Each Arm

**Figure 2:**
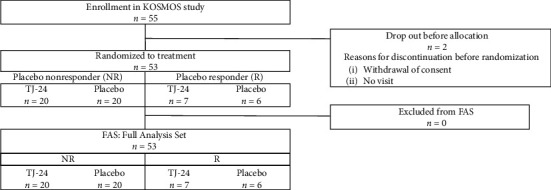
Allocation of study participants.

Figure 2 presents the allocation of participants in this study. Of the 55 individuals enrolled in the study, two withdrew before randomization (withdrawal of consent and failure to attend the study visit) and 53 participants were thus randomly allocated. None of the allocated participants missed data required for efficacy analysis, and the FAS accordingly consisted of these 53 individuals, as did the safety analysis set.

In the assessment at the end of the 4-week run-in period, 40 participants (75.5%) were classified in the NR group, and the R group included the remaining 13 participants (24.5%). Participants in both groups were randomized to receive either TJ-24 or placebo.  Table 1 presents the baseline characteristics of each arm. In the NR group, the score for waking up at night was significantly different between participants in the TJ-24 and placebo arms, but there were no other significant differences.

### 3.2. Effect of TJ-24 on Different Complaints in the Placebo NR Group

The effect of TJ-24 on participants in the NR group, who were considered less susceptible to the placebo effect, was investigated. As illustrated in Table 2, the changes of the excitability and irritability score during the treatment period, the primary outcome, decreased significantly by –3.1 ± 1.7 in the TJ-24 arm versus baseline. However, the score decreased by –2.7 ± 2.2 in the placebo arm, with no significant difference between the two arms (*p*=0.519). There was also no significant difference between the TJ-24 and placebo arms in the rate of other common menopausal symptoms including hot flashes, sweating, and sleep disturbance-related complaints (Table 2), and this was also the case for other symptoms.

Regarding secondary outcomes, an investigation of psychiatric symptoms, as assessed using HADS, revealed no significant difference over the treatment period between the TJ-24 and placebo arms. In particular, the anxiety score decreased by –0.5 ± 1.3 in the TJ-24 arm and by –0.7 ± 2.0 in the placebo arm, and the depression score increased by 0.5 ± 1.5 in the TJ-24 arm and by 0.6 ± 1.8 in the placebo arm. An investigation of sleep disturbance as assessed using PSQI also found no significant difference in the change of the total score over the treatment period between the TJ-24 and placebo arms (–1.6 ± 1.5 in the TJ-24 arm vs. –2.4 ± 2.3 in the placebo arm), and no significant difference was observed concerning the time required to fall asleep or the duration of sleep. An assessment of QOL using the SF-8 also found no significant difference between the TJ-24 and placebo arms in either the physical component summary score (3.0 ± 6.0 in the TJ-24 arm vs. 2.4 ± 6.6 in the placebo arm), or the mental component summary score (1.9 ± 7.9 in the TJ-24 arm vs. 4.0 ± 8.5 in the placebo arm).

To investigate whether baseline characteristics altered the effect of TJ-24, participants were classified by menopausal status, BMI, the duration of climacteric disorder, and prior hysterectomy/oophorectomy. Of these, menopausal status altered the effect on several symptoms. As presented in Table 3, in premenopausal women, the changes of the excitability and irritability score were significantly greater in the TJ-24 arm than in the placebo arm (–3.6 ± 1.5 vs. –2.2 ± 2.1), suggesting that TJ-24 may be effective against excitability and irritability in women who have yet to undergo menopause. In postmenopausal women, there was no significant difference in the excitability and irritability score between the groups, whereas the changes in the scores for hot flashes and sweating were significantly larger in the placebo arm than in the TJ-24 arm. There were no significant differences for any other baseline characteristics.

In line with the results in the NR group, there were no significant differences concerning various different complaints between the TJ-24 and placebo arms in the R group.

### 3.3. Detailed Analysis of the Effect of TJ-24 on Excitability and Irritability

**Figure 3:**
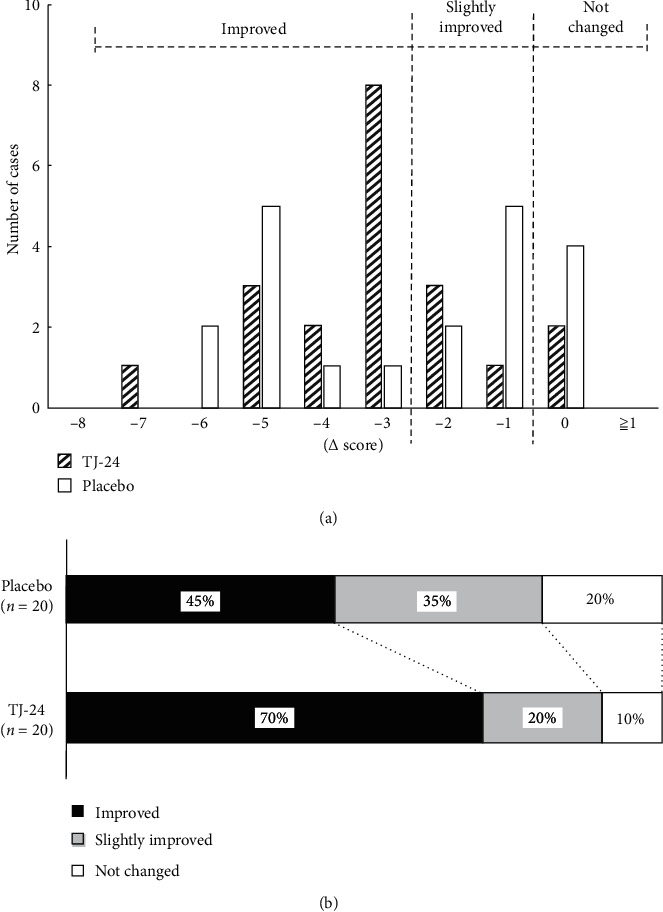
The distribution of score changes for excitability and irritability.

To analyze the placebo effect in detail, the effects of TJ-24 and the placebo on excitability and irritability were investigated in a post hoc analysis.  Figure 3(a) presents the distribution of changes in the excitability and irritability score over the treatment period. In the TJ-24 arm, the change was almost evenly distributed, peaking at –3. Contrarily, in the placebo arm, the distribution had double peaks at –1 and –5. Using thresholds of score changes of ≥3 for improvement and change of 1 or 2 for slightly improvement, the proportion of participants with improvement was 70% in the TJ-24 arm and 45% in the placebo arm, representing a significant difference (Figure 3(b)).

### 3.4. Safety of TJ-24 Administration

An analysis of the FAS found that 17 adverse events occurred in 11 participants and six adverse drug reactions occurred in four participants in the TJ-24 arm, including six cases of the common cold or influenza, and one case each of vomiting, diarrhea, cutaneous pruritus, and herpes labialis. In the placebo arm, 11 adverse events occurred in seven patients, and two adverse drug reactions occurred in two patients, including four cases of the common cold, two cases of constipation, and one case each of diarrhea and cutaneous pruritus. No event was serious. There was no significant difference in the incidences of either adverse drug reactions or adverse events between the TJ-24 and placebo arms in the NR and R groups. There were no abnormal results in the blood testing, excluding one case of slight hyperkalemia in a patient who received TJ-24.

## 4. Discussion

In Japan, HT is not widely used to treat climacteric disorder. Conversely, because of its safety, Kampo medication is more widely used, and that drug adherence is high. The medications used to treat climacteric disorder include tokishakuyakusan, TJ-24, and keishibukuryogan, and of these, TJ-24 is most widely used as a formulation of dry extract granules based on the preparation listed in the *Wazai Kyokuho*, the classical pharmacopeia of Kampo medicine. Clinical studies reported that TJ-24 improved neuropsychiatric symptoms [6, 7], vasomotor symptoms [6], and sleep disturbance [7] in patients with climacteric disorder. In basic studies, kamishoyosan was revealed to exert an anxiolytic effect through stimulation of the *γ*-aminobutyric acid A-benzodiazepine receptor in male mice [15] and socially isolated ovariectomized rats [16]. Moreover, kamishoyosan was demonstrated to exert an antidepressive effect through the 5-HT1A receptor and PKA-CREB-BDNF signaling in postmenopausal depression model mice [17]. However, few previous clinical studies conducted objective investigations from the perspective of evidence-based medicine. In a previous double-blind, randomized trial, we observed a major placebo effect, resulting in no significant difference in the frequency of hot flashes between TJ-24 and placebo [10]. In planning the present study, therefore, a study design was employed to investigate the actual effect of TJ-24. Climacteric disorder encompasses numerous symptoms and complaints, and previous randomized, controlled trials mainly focused on hot flashes or secondary depressive symptoms that may result from the presence of these symptoms and complaints. Because the results of previous open trials suggested that TJ-24 improves psychiatric symptoms, particularly irritability and insomnia, excitability and irritability comprised the primary outcome. The study design also included a run-in period to exclude women particularly susceptible to the placebo effect, aiming to reduce the impact of this effect. The intention was to ascertain the effect of TJ-24 by targeting placebo nonresponders.

Although TJ-24 significantly improved the score for excitability and irritability, the primary outcome, in the NR group, in line with our previous study, there was no significant difference in the score change between the TJ-24 and the placebo arms. However, an assessment of the proportion of participants whose score improved by ≥3 points revealed a significantly higher rate in the TJ-24 arm, suggesting that TJ-24 may have had an effect and supporting the impression of efficacy observed in clinical practice. Concerning safety, no worrisome severe adverse drug reactions or adverse events were observed, and there was no difference in the incidence of adverse drug reactions or adverse events between the study arms, indicating that TJ-24 is safe for use.

In the subgroup analyses, TJ-24 significantly reduced the score for excitability and irritability in premenopausal women. Hidaka et al. reported that TJ-24 is effective against psychiatric symptoms [6], and mental factors are known to play a major role in the perimenopausal period. In fact, it is well known that depression causes the appearance of menopausal symptoms [18]. Women with no previous history of depression are reportedly at higher risk of perimenopausal depression prior to menopause, and this risk decreases after menopause [19]. In clinical practice, it is difficult to provide HT to premenopausal women, and the fact that TJ-24 improves excitability and irritability in these women is one advantage of the medicine. This symptom is also comparatively common in Japanese women [20]. This suggests that TJ-24 may help improve QOL as a nonhormonal treatment option for perimenopausal women who cannot be prescribed HT or who do not wish to take it. Regarding sweating and hot flashes, the score change among postmenopausal women was larger in the placebo group than in the TJ-24 group, suggesting that the change was likely a placebo effect. In addition, in this study, we selected women with available excitability and irritability score and at least one menopausal symptom (hot flashes, sweating, difficulty in sleeping, and waking up at night). Therefore, the baseline sweating and hot flash score was not constant at baseline, and thus, we considered that the finding was a possible incidental result of statistical analysis.

The strength of the placebo effect may be attributable to the following reasons. The first is the problem of the criteria for placebo responders. In the present study, participants whose excitability and irritability scores improved by ≥3 points following the 4-week run-in period were considered placebo responders. The rationale for this was that placebo use is believed to improve menopausal symptoms by approximately 34% [9, 21], and thus, a score change of ≥3 points, corresponding to *a* ≥30% improvement, was considered to indicate response. In this study, 24.5% of patients were included in the R group, a somewhat low proportion, suggesting that the NR group may also have included some responders. In addition, it is possible that the placebo effect could have been more completely eliminated by setting the cutoff at ≥2 points. However, if a cutoff of ≥2 points had been used, the NR group would only have included 56.5% of participants, and securing sufficient numbers for this group would have been an issue.

Second, it is possible that the 4-week run-in period may have been too short, and the placebo effect may have been eliminated in a longer period. This point was repeatedly considered before starting this study, but from an ethical standpoint, a run-in period exceeding 4 weeks would have been inappropriate.

A third possibility is that the participants may have identified the placebo. They were not informed that they were taking a placebo during the run-in period, and they may have realized that their medication had changed after randomization. The severity of symptoms was also assessed subjectively by the participants themselves rather than by an objective index, and when the same questionnaire is used, there is a tendency to indicate that improvements have occurred.

Fourth, the participants included premenopausal women. Premenopausal and postmenopausal women may have different mechanisms of onset for menopausal symptoms. The hormone levels in these women are inconsistent, and individual differences in factors other than hormone levels may also have had an effect. In fact, in terms of the clustering of menopausal symptoms, they are believed to stabilize after menopause [22].

The fifth issue is seasonal changes. Menopausal symptoms have been reported to change with the seasons [23], and the difference between the placebo and actual drug may have narrowed at some points. However, in the present study, no adjustment was made for the timing of the investigation.

The study also had several other limitations. The first limitation was the dosage. Increasing the dose might possibly have resulted in a stronger effect. However, other studies of Kampo medication have found that increasing the dose did not influence outcomes [9], and in the present study, the normal dose under Japanese approval conditions was used. The second limitation was the sample size. As mentioned previously, increasing the number of participants might have weakened the impact of the placebo effect, and it may be necessary to reconsider the sample size. However, recruiting participants is also difficult. The third limitation was the administration period. Although there is justification for extending the protocol, particularly the run-in period, this might pose an ethical problem.

In this multicenter, randomized, double-blind, placebo-controlled trial of TJ-24 for the treatment of climacteric disorder, no difference was observed in the score for excitability and irritability between the TJ-24 and placebo arms because of a placebo effect. However, given that the proportion of participants whose score improved by ≥3 points was significantly larger in the TJ-24 arm, the safety results were good, and the excitability and irritability score improved significantly in premenopausal women, TJ-24 may represent an option for nonhormonal treatment. Further studies are warranted to further examine these issues.

## Figures and Tables

**Table 1:** Baseline characteristics of the study participants.

	Placebo nonresponder (NR)			Placebo responder (R)			NR vs. R
	TJ-24 (*n* = 20)	Placebo (*n* = 20)	*P* value	TJ-24 (*n* = 7)	Placebo (*n* = 6)	*p* value	*p* value
Age (years)	51.5 ± 2.6	51.2 ± 3.4	0.798a	52.1 ± 2.5	50.7 ± 1.4	0.231a	0.881a
BMI (kg/m2)	21.0 ± 1.9	22.9 ± 4.2	0.072a	24.4 ± 2.6	22.6 ± 3.0	0.294a	0.126a
Time from LMP (days)	750.6 ± 889.7	708.0 ± 946.9	0.884a	498.3 ± 342.5	232.3 ± 136.6	0.103a	0.175a
Menopausal status							
** **Premenopausal	11 (55.0%)	11 (55.0%)	1.000 b	2 (28.6%)	5 (83.3%)	0.103 b	1.000b
** **Postmenopausal	9 (45.0%)	9 (45.0%)		5 (71.4%)	1 (16.7%)		
Disease duration (months)	26.3 ± 20.3	21.8 ± 19.7	0.500a	27.0 ± 18.3	11.8 ± 10.0	0.099a	0.521a
Excitability and irritability score	7.2 ± 1.3	6.9 ± 1.4	0.625c	3.7 ± 1.8	3.8 ± 1.9	1.000c	<0.001c
Hot flashes score	5.7 ± 2.2	6.0 ± 2.2	0.582c	5.7 ± 2.5	4.2 ± 2.1	0.184c	0.211c
Sweating score	6.3 ± 2.1	6.7 ± 2.2	0.534c	6.3 ± 0.8	4.2 ± 2.4	0.069c	0.048c
Difficulty in sleeping score	5.3 ± 2.6	5.4 ± 2.7	0.870c	4.1 ± 2.3	4.2 ± 2.9	0.885c	0.189c
Waking up at night score	4.6 ± 2.6	6.4 ± 2.2	0.024c	6.3 ± 1.9	3.8 ± 2.9	0.131c	0.723c

^a^Student's *t*-test. ^b^Fisher's exact test. ^c^Wilcoxon's rank-sum test.

**Table 2:** Changes of scores in the modified questionnaire for climacteric symptoms in Japanese women (placebo nonresponder group).

Mean changes of scores in the modified questionnaire for climacteric symptoms in Japanese women	Placebo nonresponder (NR)		
	TJ-24 (*n* = 20)	Placebo (*n* = 20)	*p* value
Excitability and irritability	−3.1 ± 1.7	−2.7 ± 2.2	0.519
Hot flashes	−1.9 ± 2.4	−1.9 ± 3.0	0.816
Sweating	−1.6 ± 2.7	−1.6 ± 2.9	0.989
Difficulty in sleeping	−1.9 ± 2.1	−1.9 ± 2.3	0.923
Waking up at night	−1.2 ± 2.8	−1.8 ± 2.5	0.595

Data are the mean changes from baseline of climacteric symptoms at week 12 in the placebo nonresponder group.

**Table 3:** Subgroup analysis of changes of scores in the modified questionnaire for climacteric symptoms in Japanese women (placebo nonresponder group).

Mean changes of scores in the modified questionnaire for climacteric symptoms in Japanese women	Premenopausal			Postmenopausal		
	TJ-24 (*n* = 11)	Placebo (*n* = 11)	*p* value	TJ-24 (*n* = 9)	Placebo (*n* = 9)	*p* value
Excitability and irritability	−3.6 ± 1.5	−2.2 ± 2.1	0.046	−2.3 ± 1.7	−3.2 ± 2.3	0.323
Hot flashes	−2.2 ± 2.6	−0.2 ± 2.7	0.116	−1.4 ± 2.2	−3.9 ± 1.9	0.032
Sweating	−2.4 ± 3.4	−0.5 ± 3.2	0.129	−0.7 ± 1.3	−2.8 ± 2.0	0.039
Difficulty in sleeping	−1.9 ± 2.2	−0.9 ± 1.8	0.363	−1.9 ± 2.0	−3.1 ± 2.4	0.416
Waking up at night	−0.5 ± 3.1	−1.2 ± 2.5	0.894	−1.9 ± 2.3	−2.6 ± 2.5	0.533

Data are the mean changes from baseline of climacteric symptoms at week 12 in the placebo nonresponder group.

## Data Availability

The data used to support the finding of this study are available from the corresponding author upon request.
